# The Life of John Walker, M.D. Late Director of the Royal Jennerian and London Vaccine Institutions

**Published:** 1831

**Authors:** 


THE LIFE OF JOHN WALKER, M.D. late Director of the Royal Jennerian and London Vaccine Institutions. By *John Epps*, M.D. Director of the above Institution, &c. Octavo, pp. 342. London, 1831.

THE name of John Walker is familiar to the ears of the whole profession; and the person of that remarkable individual will be remembered by all those who have been educated at the Borough schools during the last quarter of a century, in consequence of the eccentricity of manners which he displayed in the Physical Society, where he was a very constant attendant —and not seldom the source of considerable merriment. His unwearied zeal, not to say enthusiasm, in the cause of vaccination, and of humanity in general, has also familiarized him with, and endeared him to the hearts of all philanthropists in and out of the profession. The life of this singular personage has been more eventful than that of most medical men—and as delineated in this volume by the able pen of his friend and successor Dr. Epps, forms one of the most amusing and instructive pieces of medical biography in the English language. The interest and the merits of the book would be sufficient passports to public patronage—but there is a still higher consideration in question. The publication is brought forth for the benefit of the widow. The work is dedicated to the WORLD, for whose good the life of Walker was spent and devoted—and it is to be hoped that the WORLD will make some return by promoting the welfare of his surviving partner.

From a long acquaintance with Dr. Walker, we can well believe Dr. Epps, that there was, " amidst the numerous papers left by him, a chaotic confusion." It must have cost the biographer no small labour to draw order out of chaos on this occasion. But we shall now proceed to exhibit a rapid sketch of this most extrordinary man's life, recommending, in the strongest terms, the whole work to our readers, convinced as we are that they will be well repaid for their time and money, by the treasure of anecdote and the fund of moral and humane sentiment which this volume contains.

Dr. Walker was born in Cockermouth, county of Cumberland, on the 31st July, 1759, where he and the late celebrated Dr. Woodville were school-fellows. Young Walker was the idlest of boys at his tasks—the most active in play. Finding that the master generally took a nap while he was saying his lessons, he hit on the plan of getting a few lines at the beginning and end, thus calculating on non-detection of all the intermediate errors.

> " Cultivating this dependence, he learned generally only two or three of the first lines, and a few at the end of the lesson. Before completing the few he knew, the master began to nod. Young Walker kept his eye fixed upon the sleeper, keeping up, at the same time, a humming sound, without articulating a syllable, till the master, giving a greater nod than usual, awoke, when the young rogue repeated the last line of his task and went to his seat. When the honest pedagogue was sufficiently on the alert, the deficiency was detected, and Walker, flagellated, was sent to his form." 5.

Walker's father was a smith and iron-monger in Cockermouth, and after leaving the grammar-school, the son, on mingling in the fracas of the bellows, the hammers, and the anvils, was soon adorned with the insignia of his occupation, the leather-apron, consecrating cheerfully his power to the forge, by inscribing on its entrance the Roman poet's description of the forge of Cyclops :—

> " The hissing steel is in the smithy drown'd."

Five years passed away in this manner, the young Walker not doing much, however, of the laborious drudgery, but employing himself a good deal in the mechanical construction of scale-beams, locks, and other pieces of machinery not very complicated in their nature. A temporary residence at his father's house of a broken down tradesman from Dublin inspired young Walker with an ardent thirst for drawing and engraving, in which branches he made some progress. But, in fact, though Walker had a smattering of many things, he had no perfect knowledge of any thing, and found himself, at the age of twenty, " the most helpless of creatures." At this time a sudden resolution seized the mind of young Walker—to enter as a common sailor on board a *privateer!* His father gave him a quantity of scale-beams to dispose of in Dublin, where the privateer lay, and away went the youth in quest of adventures. By the time he reached the Irish metropolis, in 1779, the privateer was captured, and there was a hot press prevailing for men-of-war's men, a situation which Mr. W. did not seem to relish. He therefore settled in Dublin—sold his scales—became acquainted with some of the young Dublin rakes, and was engaged in many a midnight row and revel. At length he placed himself under Esdale the engraver, and spent four years in the pursuit of engraving, and Walker's Hibernian Magazine for 1780-1-2-3, contain many plates of his execution. Walker excelled in landscape; and while pursuing his studies in this line, he bettered his acquaintance with the dead languages, in which he had hitherto made but little proficiency.

With that versatility and love of change which characterized his whole life, Walker now suddenly turned schoolmaster, and by dint of perseverance, got up a school containing 100 scholars. Walker now became a Quaker in sentiment and religious views, but the Society of Friends would not admit the young candidate within their pale, fearing that he had too much learning to be religious. In the midst of his scholastic struggles, he turned author, and published his System of Geography and his Gazetteer, which made some noise in their day. While constructing these works he was so poor that he could scarcely afford to buy candles whereby to study after school-hours! In 1792 he made the attempt to publish, on a large scale, the second edition of his Geography and Gazetteer, which did not turn out successful, and obliged him to give up his school and come to London, after ten years spent as a pedagogue—one of the most irksome occupations, we should think, to which man was ever doomed!

In London, Walker became acquainted with a navy-surgeon unemployed, and here he took a violent predilection for medical studies—entered himself as a student at the Borough hospitals, though on the wrong side of thirty years of age, and spent three years in the ardent pursuit of professional knowledge. Always desirous of change he went to the Continent in 1797, and visited Paris in the garb of a Quaker, where he was civilly treated, and permitted to ramble about unmolested, and indeed to the great entertainment of the mercurial Parisians. In 1799 he obtained a degree at Leyden, of Doctor in Medicine—and soon afterwards returned to England, where he became united to the object of his affections at Glasgow. Dr. Walker maintained that marriage was a civil contract—not a religious ceremony; and by this doctrine gave still greater offence to the Quakers, who still kept him out of their brotherhood. He now took up his residence in MODERN ATHENS, and studied there for some time, making the acquaintance of many eminent professors, among others, Dugald Stewart, to whose table Dr. W. was often invited.

In 1800 he was invited by Dr. Marshall to accompany him to Naples, for the purpose of introducing vaccination into the Neapolitan states. He took passage in the Endymion frigate, and the voyage furnished the eccentric Quaker with many topics for remark—many subjects of wonderment and admiration. His description of beating to quarters, and clearing for action, in the night, is not bad; though not quite so spirited or faithful as Dr. Epps imagines. It is, however, quite graphic enough for general readers. The Doctors remained at Gibraltar three weeks, introducing vaccination among the military and civil population. From Gibraltar the vaccinators proceeded to Minorca in a Florentine frigate. At Mahon the vaccine virus failed, and they were obliged to send back to Gibraltar for fresh virus, which succeeded. They then visited Malta, and there introduced vaccination. After a short visit to Naples and Sicily, Dr. Walker proceeded with the English fleet for Alexandria, leaving Dr. Marshall at Malta. Dr. W. was present at the landing of the troops and at the battle where Abercrombie lost his life. He first directed his attention to the relief of the wounded—and then prosecuted his vaccine labours with success. The Pacha of Rosetta made him a present; but it appears that he was scurvily treated by the British Government, and actually wronged out of money which he laid out for the use of the sick. But mismanagement and misrule were too much the order of the day at that time. They are not very likely to return.

We imagine that by proper documents and proper representations, the widow would still receive the trifling sum which ought to have been awarded.

An amusing anecdote is here related of the eccentric doctor.

"Dr. Walker had, as the reader will have perceived, the courage to be singular. He allowed, while in Egypt, his beard to grow, so as to look very like a learned Jew. One of the young and thoughtless friends of his mess drew in chalk the French insignia, so hateful to the Turks, the *fleur-de-lis,* on his big white hat. Rising from dinner, the hat was put on, and, falling into one of his musing moods, the bearded sage wandered through Cairo without any uniform. Conceive his astonishment, when in the midst of his meditations, some Turkish soldiers fell upon him with great violence, believing—notwithstanding all his assertions to them, in an unknown tongue, that he was 'Inglese'—him to be a Frenchman. And let Britain be ashamed of her sons (many of them now, it is true, no longer able to abuse the name of their God), when they read the fact, that the Turks, in order to satisfy themselves whether Dr. Walker was or was not a Frenchman, uttered the oaths, 'God d——,' 'by God,' inferring that, if the subject of this memoir was an Englishman, he would understand a language which they had heard so generally used. Dr. Walker, horrified at the oaths they uttered, especially as coming from strange lips, instead of smiling assent, as they expected he would were he 'Inglese,' shook his head. This they understood as a mark of his not understanding them, and, consequently, that he could not be an Englishman! And the Arnhaut, who had applied this test, smiled triumphantly on his companions at his skill in detecting the Frenchman. They, therefore, seized him and took him to prison to the citadel. The prison doors were before him; and Dr. Walker, thinking that he might be put into one of the dungeons below, where he would, most likely, be never more thought of, gave himself up as one no more to enjoy the delights of home and its social pleasures. Much to his happiness, however, they bade him ascend a staircase, running their bayonets into him and knocking him with the butt-ends of their muskets behind as he ascended. While thus maltreated, and in such peculiar peril, an English patrole happened to be passing, who informed the commanding officer who the bearded philosopher—imagined by the soldiers to be a French *savant*—was, and Dr. Walker once more experienced the sweets of liberty, after enjoying the delicious dish called *killaw* with the officer and the Mussulmans, who accommodated the unbeliever with a low stool and wooden spoon, while they sat cross-legged and with naked hands helped themselves to the savoury mess." 58.

With all due veneration for the Doctor's rigid morality, we conceive that he would have been a very great fool to have sacrificed his life rather than utter the only words by which the ignorant Turkish soldier could distinguish him from the then enemy of their country.

Most men would have shaved off beards that were likely to bring them into so many difficulties. Not so Dr. Walker. "His beard singularised him; and he loved to be noticed." He entertained also some absurd notion that this ancient ornament protected him from certain cutaneous diseases of the climate. By sleeping in the open air, one night, he was seized with a paralytic affection, which made him anxious to return to his native land. After several personal adventures, not of a very formidable nature to those who have journeyed in foreign lands, but which must have appeared very interesting to a cockney-traveller, Dr. Walker embarked in the El Carmen, and returned to England.

"Delightful indeed did the green fields of England appear to the subject of this memoir, and he would willingly have taken possession of the first boat that came alongside, when anchored at Spithead, to hasten ashore." 68.

But the quarantine was put in force against him and all his companions except Sir Sidney Smith and Col. Abercrombie, who were immediately liberated, in consequence of their despatches, and allowed to proceed to London. The worthy Doctor inveighed against this "distinction of persons;" and certainly it is vexatious as well as ridiculous in a medical point of view—but the Doctor was old enough to know that, in all countries, there is one law for the powerful and another for the weakly—that CONTAGION is only communicable through particular channels—and that the quarantine doctors could prove, by the most irresistible arguments, that the plague might lurk in the *beard* of a WALKER, though quite incapable of taking up its quarters in the *whiskers* of a SMITH or an ABERCROMBIE. The poor Quaker was an infant in medical politics!

After a fortnight's repose at Stonehouse, Dr. Walker repaired to London, and in August 1802 commenced the practice of vaccination at the house of the late Mr. Fox, the celebrated dentist. He soon formed the idea of a public institution. This was soon effected, and called the Royal Jennerian Society. It is a curious fact that his present Majesty, then Duke of Clarence, was one of the first patrons and supporters of the vaccine establishment. Dr. Walker was elected resident inoculator; but some intrigues being called into play and parties formed against him, he resigned the office. The Doctor now appeared to be in a worse state than ever; but some of his friends laid their heads together, and a new society, the LONDON VACCINE INSTITUTION, was the result. It was carried on in Salisbury-court, where all the patients of the old institution flocked. In this state of things, Mr. Rose brought in a bill in parliament for the support of the old or Jennerian Institution, and £.3000 per annum of the public money was voted for that purpose. The life of Dr. Walker, after this, is the history of vaccination, and this we must pass over. The following passage will shew the activity of the Doctor to the hour of his death.

"Dr. Walker, in other words, was the monarch at the vaccine stations. His was the despotism of knowledge; and he delighted in the exercise of this kind of despotic power as much as the autocrat of the Russians does in his. Thus gratified, and impelled likewise by a sense of his duty, by the delight of doing good, and also by the pleasure of cherishing a cause of which he was the principal support, it is not a matter of wonder that he should have never missed a day, from the time when he was appointed till within a week or two of his death, in visiting the stations. It is becoming that these stations, at which he attended, should be noticed, in order to shew to the public the immense amount of service he contributed to the general good. At nine a. m. Dr. Walker was to be met with at 215, Strand; at quarter past nine, at 337, Strand; at half-past nine, at 29, Haymarket; at a quarter to ten, a. m., 27, Lisle-street; at ten, a. m., at 3, Broad-street, Bloomsbury; at a quarter past ten, at 144, High Holborn; at half past ten, at 63, High Holborn. From that station he went to one of the principal stations, at 1, Union Court, Holborn Hill (still retained by the Society), at eleven; from this he proceeded, at about a quarter to twelve, to 4½, Salisbury Court, Fleet-street, and then returned to his own house, at 6, Bond Court, Wallbrook, where he vaccinated at two, p. m. Besides these journies, on every Monday, he went to the vestry of St. John's Church, Horsley-down, kindly granted for the use of the Society; thence to the Lancaster Royal Free School, 5, Thomas-street, Borough Hospitals; and thence to the South London Dispensary, No. 1, Lambeth Road.

Such was the life of this man of benevolent industry. Day after day he went his round. Sunshine or rain, it mattered not. Vaccination was the longing of his soul; and nothing was sufficient to draw him from his course. Even the days of the annual meeting he did not neglect the stations, although staying a shorter time than usual at each. On such days he always put on a new white hat to meet the Lord Mayor, who, by virtue of his office (being President of the London Vaccine Institution), is chairman on the occasion." 125.

Dr. Walker, however, could not last for ever. The organic machine was beginning to shew that it could not much longer contain the immortal soul. In the month of February, 1830, he was suddenly awoke to a consciousness that he had passed the usual range of human existence (70) and instantly came to a resolution to prepare for death—a preparation which, no doubt, hastened that event. When he determined on any thing, he could not rest till it was accomplished. He wanted some shelves constructed in his cabinet, for the arrangement of its multifarious contents. He went for the carpenter himself, and even carried home the wood on his own shoulders. By this effort he was exhausted—complained of a pain in his side—and a previous cough was increased. He was a sceptic as to all remedies in sickness, and would take no medicine. He got worse; but still went his daily rounds. In the month of June (21st) he went his last round in a cabriolet. He then took to his bed, would take no medicine, and died on the 23d of the same month. In a report of the London Vaccine Institution, ample justice is done to the merits, the zeal, and the unwearied exertions of this singular and eccentric character who, "for upwards of a quarter of a century, never omitted one lawful day going his rounds to the numerous stations of the institution."

The mass of papers, containing observations, reflections, and anecdotes picked up on his travels, have furnished materials for several chapters in Dr. Epps' Biography, all of which will be read with much curiosity. Thus the sixth chapter exhibits Dr. Walker in Paris during the revolution, and discloses many curious scenes of that stormy event. He there became intimate with Tom Paine, Napper Tandy, Thomas Muir, and other distinguished characters that figured on the theatre of the young republic. Of Tom Paine he seems to have entertained a most exalted opinion. The following passage will afford a good specimen of the deliberate JUSTICE which we are to expect under republican institutions, should they again become the order of the day—which is more than probable.

"Among Dr. Walker's notes is a reference to the proceedings of the council of FIVE HUNDRED, in regard to newspaper editors, proceedings showing that though the members were republicans in theory, they were not so in practice.

The council met in the Odeon Theatre. The report of the committee appointed to investigate the character of periodicals was received, and, on the same, numerous individuals, including proprietors, printers, publishers, editors, &c., of opposition papers, were sentenced to deportation to the unhealthy climate of Cayenne, amid the acclamations of the attendants. With a list of nearly thirty different journals, which the committee had reported as anti-republican, the president called out the titles, one by one, with the charge, 'Ceux qui veulent qu' ils soient deportées qu'on s'elevent,' when the members generally made a little move, as if going to stand, and the president pronounced—" Ils sont deportées.' At length the rapidity of such procedure was not in conformity with the organic powers of the president, who called out, on giving a title, 'S'il n'y a point de reclamation ils sont deportées.' All sitting still, the president added—'Ils sont deportées;' and the secretaries recorded their sentence of unlimited banishment.

Sometimes a member rose in his place. The council occupied the pit. The member rising thus addressed the president:—'Citoyen, president, je demande la parolè.' 'Vous l'avez, citoyen,' replied the high officer. The member then stated that he knew well the proprietor and conductor of the journal in question. 'C'est un brave homme, un excellent citoyen, un vrai republicain.' The member further represented that it must have been from some mistake that an anti-republican article had appeared in its pages; that the editor was, perhaps, ill, or out of town, and concluded with demanding that the paper be erased from the list of those proscribed. The member having finished, the president added, 'Ceux qui veulent que les noms des proprietaires, conducteurs, &c. du papier soient erasees qu'on s'elevent.' 'Ils sont erasees.' The pleasing words were pronounced, and thus many individuals at once escaped transportation. This example given, other members took courage, rose in their seats, and succeeded in saving their friends from banishment. However, mercy was not a characteristic of many of the most declamatorily influential of the council. Agalot, Jean de Brie, and others, rushed towards the tribune and prevented the further extension of mercy. They represented that the aristocratic journalists are the greatest of all enemies to the republic; that through these the royalists were emboldened to rebel; that had not they (the council) struck the decisive blow, and seized the conspirators in their midnight machinations at the manege of the Thuilleries, and even at the Luxembourg, breaking up at once the secret confederacy, in twenty-four hours more the republic would have ceased, and we all should have been hanged. This appeal to the feelings occasioned the bursting forth of loud shouts 'Vive la Republique,' and the sentences of deportation were hurried on as before." 139.

The political opinions of the Doctor himself were unequivocally democratic; but his religious tenets, or at least the tenets of Quakerism, which he outwardly and perhaps inwardly embraced, induced him to take no active part in politics of any kind.

It is in the 8th and three succeeding chapters, however, that we are more particularly made acquainted with the personal character of Dr. Walker. These chapters will afford considerable amusement as well as instruction to the readers of the volume. We can only glean a few particulars from these chapters.

The attachment of the Doctor to his wife and to his native home are very forcibly and feelingly illustrated by various traits and memoranda which are here brought to light. The simple Quaker appears to have paid little or no attention to his own pecuniary concerns—nor even those of the institution, whose prosperity he had so much at heart. Although he was often requested to vaccinate the children of the affluent, he would seldom go to them, and rarely received any remuneration for services performed. The only thing in which he was extravagant was PRINTING. "Every thing he wrote must be printed; and if not relating directly to the Institution, he printed the same at his own expense." It need hardly be remarked that his publications were a losing concern, as the following letter to his brother-in-law, Mr. Bowman will testify.

"Dear John,—That I should hardly ever have spoken to thee on my financial affairs, as growing out of the Vaccine Institutions, may have excited thy surprise. While from time to time thou hast helped my poor frugal, striving Annie to meet payments, which the poor managers' advances 'always on account,' did not enable me to do, thou must wonder on things not coming round from year to year, so as to give me something like a proper provision.

"I am sorry to send to thee for liquidation the inclosed demand of 23*l*. 14*s*. 6*d*., for all my late literary labours. Purchases leave me at this moment without the means of paying so many shillings, I may truly say pence, as the amount of pounds in the bill." 209.

His Quaker maxim of keeping his hat on at table, and in all companies, subjected him to many inconveniences. On board the FOURDROYANT line-of-battle-ship, in the Mediterranean, he was taunted unfeelingly by the officers of the mess, and still more so by the concerted chaplain of the ship, so that he withdrew from the ward-room table, and was nearly starved by so doing!

"The opposition became at last so general, from the intimate connexion subsisting between the officers of a mess, that his own friends were influenced, and requested him to give up his 'singularities.' The lordly priest, the conceited chaplain of the ship, who acted as president at the table, said to Dr. Walker, 'you insult me in the chair.' The captain expressed his wish to interfere. Dr. Walker requested him not to do so, as he would leave the mess, and look out for a passage in another ship. Not having any regular supply of provisions, not being willing to receive from the mess, and not being able to buy any food, he suffered much in the performance of what he considered duty from the pains of hunger, so much so, that 'a hard and old bit of biscuit, a raw chesnut, or any thing falling in my way, that,' he adds, 'I knew would furnish chyle to the system, I have devoured with avidity.' This state continued upwards of a week, and he found that 'the last seven days had given him more room in his clothes than he remembered to have felt since he left England.' Happily there was one Hibernian, a warm-hearted descendant of his ancestor the Duke, who lost his life in crossing the Boyne water, who said he could not allow the Doctor to starve in the ship, and begged him to use his cabin and whatever it contained. His larder, however, was not that of an epicure; a dried tongue, a few anchovies freshened with honey (comb and all) afforded the subject of this memoir, as he himself wittily observes, 'a repast from both the animal and vegetable departments of creation.' At length the valiant transport received the conscientious Quaker, who, having taken a hearty dinner on the day of his departure, found that his stomach, from the delicacy induced by semi-starvation, would not retain its contents, a physiological fact of some practical importance. These events happened in Marmorice Bay." 224.

Dr. Epps conceives that this course of action, on the part of the Doctor, was not the result of "obstinate bravoism, but of conscientious integrity." We agree with the biographer, as far as the individual is concerned. But we cannot help considering this obstinate adherence to a ceremony so general in the world, and so purely void of even subserviency, much more of guilt, was a very foolish weakness or prejudice on the part of a man who chose to mix with the world. It was a keen and just remark of Plato, who told Diogenes, engaged in ridiculing his dress, that "there was as much pride in the rags of Diogenes as in the dress of Plato." The Doctor was outrageous, because the Duke of York ordered the soldiers to present arms on the passing of the Host, in Catholic countries. There is not much danger of this ceremony being very long in use!

It appears that Dr. Walker was not less sceptical in religion than in physic. He disbelieved in the divinity of Christ—"he did not believe the Holy Scriptures to be divine." He disregarded all revelation, "believing that the light that lighteth every man that cometh into the world is quite sufficient for every moral and spiritual purpose." Such being the Doctor's creed, or rather scepticism, we do not much wonder that the Quakers refused him admittance within their pale:—we wonder, too, what could induce him to seek admittance into their society! The following is Dr. Epps' remark on this important subject.

"And here the writer must state that, in recollecting that the subject of this memoir was a sceptic, and knowing the fact that many good men of thinking minds are in the same unhappy condition, he cannot withhold the fact, of which he is certain, that established systems of church government, necessarily corrupt, and mimicry imitations of these systems by those called Dissenters, more or less impure, are the cause of a great part of the cold infidelity of modern times. Benevolent and thinking beings see nothing of the religion of Christ in men making a trade of religion. In the hierarchy of episcopalianism or the kirk of Scotlandism, they see nothing of the simplicity of Paul and his friends; and in the little popes of dissenting congregations they behold the exhibitions, although on a miniature scale, of a certain individual who lives on the banks of the Tiber." 251.

We shall conclude with one more extract from Dr. Epps, since it conveys a spirited characteristic outline of the moral and physical man whose life has been portrayed.

"In stature, Dr. Walker was about five feet seven inches and a half. His features were long and prominent, especially the nose and the chin. His eyes were large; also his eye-brows. His forehead high; his hair dark brown, which he combed back straight like the patriarchs of old. His bones were large, and were prominently marked from the spare habit of his body. He wore a white beaver broad-brimmed hat; large broad-tail coat, the pockets full of papers, a portfolio under his arm.

He was very moderate in his meals; enjoyed his tea much, and often referred to the love of taking this delightful beverage. For the last fifteen years of his life he never took any malt or spirituous liquors, being convinced, from the rapidity of the recovery of the Mussulmans whom he attended at Alexandria, under their wounds, that the constitution of individuals taking water is in the most healthy condition. He found that he was less liable to take cold from exposure to rain and other commonly considered causes of cold, than when taking malt liquor. He partook of animal, as well as vegetable, food. The day he died, he asked for the former, in the request, 'Annie, bring me some *animal fibre.*'

He often sat up very late at night writing, being a contributor to a great variety of periodicals, which, it is hoped, will repay the debt of gratitude in the most efficient way: for Dr. Walker never was a paid scribe.

Such was this extraordinary man. He knew human nature well. He did not seem to take any notice, but was always noticing. He scorned being influenced by trifles. The laugh of ignorance he did not regard; and the finger of contempt he did not observe. He felt pleasure in his own ways; and no displeasure of others could alter him. 'John Walker could,' as his friend Mr. Cordell said in a speech to his memory at the City of London Tavern, at the Anniversary meeting for 1831, 'never be forced; but could always be led by a silken cord.' There was in him a stream of benevolence that would have fertilized wherever it flowed, had not its course been too rapid and impetuous. He was the apostle of vaccination. He went out, in truth, without scrip or purse. His life was a continual exertion for the happiness of the human race; and, though he felt a little pride in these exertions, let us forget this in the benefits which, from his labours, society now experiences." 292.

Dr. Epps has performed his task well—and sincerely do we hope that the public will aid the author in his benevolent exertions for the helpless and surviving widow, by purchasing extensively the work which we have just closed.

